# No difference in hepatocellular carcinoma risk in chronic hepatitis B patients treated with tenofovir vs entecavir: evidence from an updated meta-analysis

**DOI:** 10.18632/aging.202573

**Published:** 2021-02-26

**Authors:** Jie Yuan, Yang Peng, Fa-Bao Hao, Ya-Qin Wang, Chun-Rui Wang, Guo-Chao Zhong

**Affiliations:** 1 Department of Surgery, Maternal and Child Health Hospital of Yongchuan, Chongqing, China; 2 Department of Geriatrics, The Fifth People’s Hospital of Chengdu, Chengdu, China; 3 Department of Neurosurgery, Qingdao Women and Children’s Hospital, Qingdao University, Qingdao, China; 4 Department of Children Healthcare, Maternal and Child Health Hospital of Yongchuan, Chongqing, China; 5 Department of Infectious Diseases, Institute for Viral Hepatitis, The Key Laboratory of Molecular Biology for Infectious Diseases, Chinese Ministry of Education, The Second Affiliated Hospital of Chongqing Medical University, Chongqing, China; 6 Graduate School, Chongqing Medical University, Chongqing, China; 7 Department of Hepatobiliary Surgery, The Second Affiliated Hospital of Chongqing Medical University, Chongqing, China

**Keywords:** tenofovir, entecavir, hepatocellular carcinoma, meta-analysis

## Abstract

Whether tenofovir disoproxil fumarate (TDF) is superior to entecavir in reducing hepatocellular carcinoma (HCC) risk among treatment-naïve chronic hepatitis B (CHB) patients remains controversial. We aimed to clarify this controversy. Several databases, including PubMed and Embase, were retrieved through November 2020. Cohort studies comparing the effectiveness of TDF and entecavir in reducing HCC incidence among treatment-naïve CHB patients were included if they reported multivariable-adjusted or propensity-score-matched risk estimates. A random-effects model was used to pool hazard ratios (HRs). Thirteen cohort studies, involving 4097 HCC cases and 80202 CHB patients, were included. Multivariable-adjusted meta-analysis revealed no significant difference in HCC incidence between TDF and entecavir groups (HR 0.86, 95% confidence interval 0.72–1.04), which was consistent with propensity-score-matched meta-analysis (HR 0.83, 95% confidence interval 0.66–1.03). Subgroup analysis showed that the observed similarity of TDF to entecavir for HCC prevention persisted in studies with follow-up length of ≥4 years but not in those with follow-up length of <4 years (*P*_interaction_<0.01). In conclusion, TDF is similar to entecavir in reducing HCC incidence among treatment-naïve CHB patients. Heterogeneous results of included studies may result from their disparity in follow-up length. Our findings should be treated with caution and need to be further confirmed.

## INTRODUCTION

Chronic hepatitis B (CHB) infection remains a serious public health problem worldwide, with around 290 million individuals carrying hepatitis B virus (HBV) [1]. Continuous replication of HBV is a major driver of progression from CHB to cirrhosis and hepatocellular carcinoma (HCC) [2, 3], therefore, long-term antiviral therapy for persistently suppressing HBV replication has been widely used to prevent disease progression in CHB patients.

Tenofovir disoproxil fumarate (TDF) and entecavir, two nucelos(t)ide analogues with high genetic barrier to HBV resistance, are first-line antiviral agents for CHB according to current international practice guidelines [4–6]. Both agents have been shown to be effective in reducing HCC incidence among CHB patients [7–9]. However, whether they differ in the degree of improving such an outcome remains unclear [10–19]. A few meta-analyses on this topic had been published [20–30], but they presented inconclusive results. Specifically, most of them found that TDF was associated with a reduced risk of HCC compared with entecavir [20–28], while two contemporary meta-analyses failed to observe the putative superiority of TDF over entecavir in reducing the risk of HCC [29, 30]. More importantly, published meta-analyses could be severely affected by confounding bias, as they combined unadjusted risk estimates with adjusted risk estimates; also, they did not perform subgroup analyses to identify the potential effect modifiers for the comparative effectiveness of TDF vs entecavir in the prevention of HCC (e.g., cirrhosis). In addition, several subsequent observational studies consistently found that there was no significant difference in HCC incidence between TDF and entecavir groups [31–33]; hence, it is essential to perform an updated meta-analysis to determine whether the results of previous meta-analyses persisted after including newly published studies.

Therefore, we performed this study to investigate the comparative effectiveness of TDF vs entecavir in reducing HCC incidence among treatment-naïve patients with CHB.

## RESULTS

### Literature search

**Figure 1:**
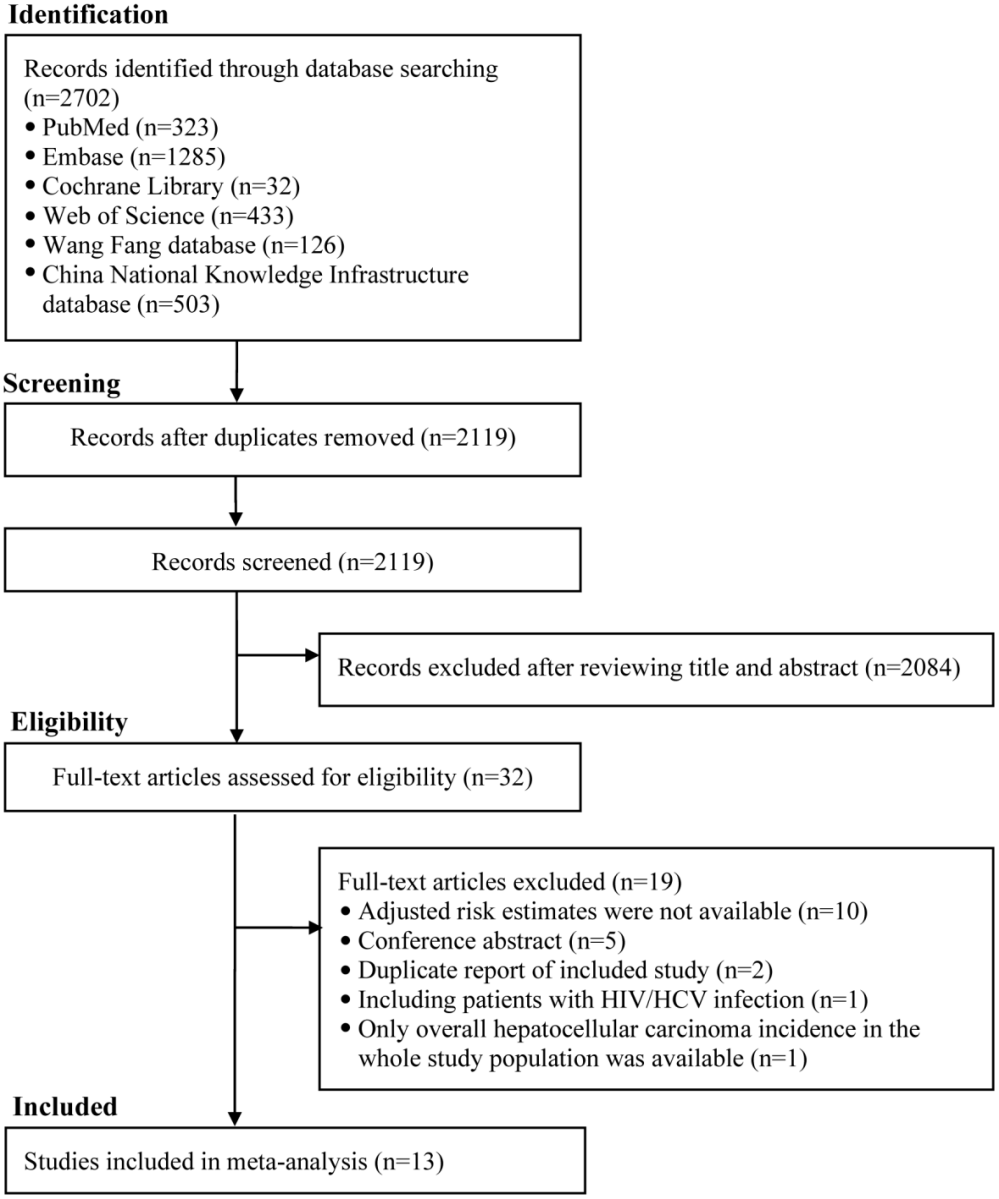
**The flowchart of identifying relevant studies.** HIV, human immunodeficiency virus; HCV, hepatitis C virus; HCC, hepatocellular carcinoma.

The literature retrieval initially identified 2702 citations. A total of 2119 citations remained after removing duplicates. After scrutinizing titles and abstracts, a total of 32 citations were thought to be potentially relevant. Nineteen citations were excluded after carefully reading the full text (Supplementary Table 1 shows the primary reason for exclusion). Thus, a total of 13 studies involving 14 cohorts were included (Figure 1).

### Study characteristics and quality assessment

Main characteristics of included studies are shown in Supplementary Table 2. These studies were published between 2018 and 2020. Eight studies were conducted in Korea [10, 11, 13, 15–17, 31, 33], one in the USA [19], two in China [14, 32], one in the Europe [18], and one in the USA, China, Japan, and Korea [12]. The sample size of included studies ranged from 404 [33] to 29350 [14], with a total of 80202 patients. The follow-up duration varied from 3.0 years [15] to 7.1 years [18]. HBV DNA levels were somewhat lower in the TDF group than in the entecavir group in nine out of 13 included studies [10–15, 19, 31, 33]. The quality of included studies was generally good, with an average score of 6.9 stars (Supplementary Table 3).

### Meta-analysis

**Figure 2:**
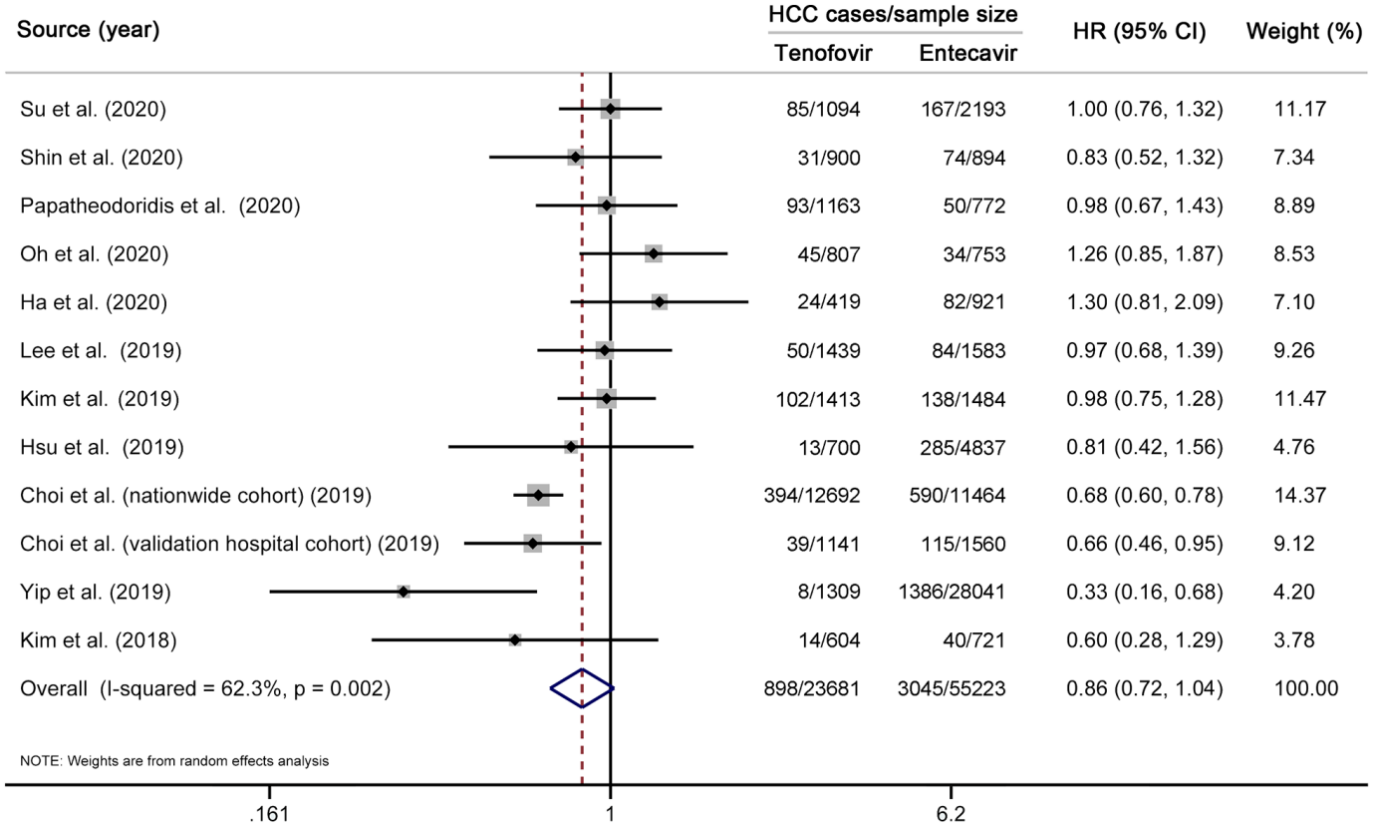
**Multivariable-adjusted meta-analysis comparing the effectiveness of TDF vs entecavir in reducing HCC risk.** Note that this meta-analysis is based on multivariable-adjusted risk estimates. The squares represent risk estimate of each included study, with the area reflecting the weight assigned to the study. The horizontal line across each square represents 95% CI. The diamond represents the pooled risk estimate, with width representing 95% CI. TDF, tenofovir disoproxil fumarate; HCC, hepatocellular carcinoma; HR, hazard ratio; CI, confidence interval.

**Figure 3:**
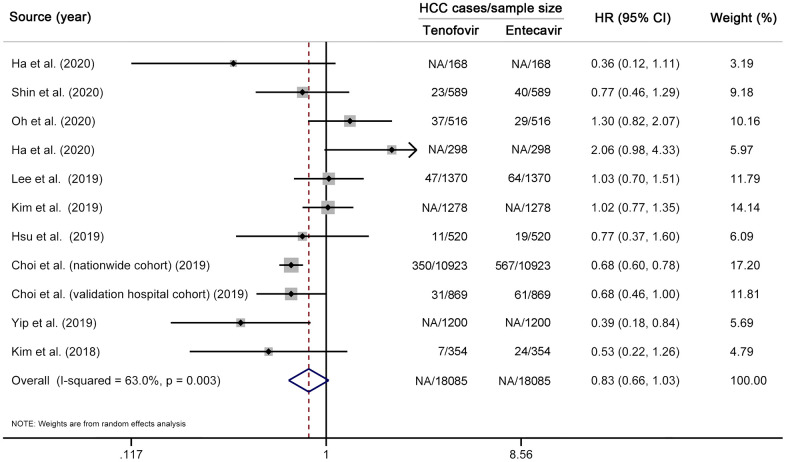
**Propensity-score-matched meta-analysis comparing the effectiveness of TDF vs entecavir in reducing HCC risk.** Note that this analysis is based on risk estimates from propensity-score-matched analyses. The squares represent risk estimate of each included study, with the area reflecting the weight assigned to the study. The horizontal line across each square represents 95% CI. The diamond represents the pooled risk estimate, with width representing 95% CI. TDF, tenofovir disoproxil fumarate; HCC, hepatocellular carcinoma; HR, hazard ratio; CI, confidence interval.

We first performed a multivariable-adjusted meta-analysis. A total of 11 studies (12 cohorts) [10–19, 31], involving 3943 HCC cases and 78904 CHB patients, were included. No significant difference in the risk of HCC was found between TDF and entecavir groups (HR 0.86, 95% CI 0.72–1.04, *I*^2^=62.3%, *P*_heterogeneity_ < 0.01) (Figure 2). We then performed a propensity-score-matched meta-analysis, which included ten studies (11 cohorts) [10–17, 31, 33] with 18085 matched pairs (Supplementary Table 4). Similar to the results of multivariable analysis, no significant difference in HCC incidence was found between TDF and entecavir groups (HR 0.83, 95% CI 0.66–1.03, *I*^2^=63.0%, *P*_heterogeneity_ < 0.01) (Figure 3). Finally, for comparison with the results of multivariable-adjusted and propensity-score-matched meta-analyses, we performed a meta-analysis of unadjusted risk estimates. Based on 12 studies (13 cohorts) [10–19, 31, 33], HCC incidence was found to be significantly lower in the TDF group than in the entecavir group (HR 0.75, 95% CI 0.60–0.95, *I*^2^=80.9%, *P*_heterogeneity_ < 0.01) (Supplementary Figure 1).

### Subgroup and sensitivity analyses

Subgroup analyses showed that the similarity of TDF to entecavir for HCC prevention was not modified by study location, study source, study setting, cirrhosis, and the exclusion of patients with decompensated cirrhosis (all *P*_interaction_ > 0.05) (Table 1). However, such a similarity was modified by follow-up length (*P*_interaction_ < 0.01). Specifically, no significant difference in risk reduction of HCC was found between TDF and entecavir groups in studies with follow-up length of ≥ 4 years (HR 1.01, 95% CI 0.88–1.17), while TDF was found to be associated with a reduced risk of HCC than entecavir in studies with follow-up length of < 4 years (HR 0.68, 95% CI 0.54–0.86).

**Table 1:** Subgroup analyses on the comparative effectiveness of tenofovir versus entecavir for reducing hepatocellular carcinoma risk^¶^.

**Subgroup**	**N**	**HR (95% CI)**	***I***^2^ **(%)**	***P*** ^†^	***P*** ^‡^
Study location					
Korea	8	0.88 (0.72–1.07)	64.4	0.01	0.64
Non-Korea	4	0.76 (0.50–1.16)	63.1	0.04	
Study source					
Population-based	4	0.82 (0.64–1.05)	56.7	0.07	0.79
Hospital-based	8	0.86 (0.68–1.10)	59.0	0.02	
Study setting					
Multicenter	7	0.85 (0.66–1.08)	72.3	<0.01	0.95
Single-center	5	0.86 (0.67–1.11)	35.2	0.19	
Cirrhosis					
Yes	9	0.81 (0.67–0.97)	31.9	0.16	0.09
No	5	1.15 (0.87–1.53)	0.0	0.64	
Exclusion of patients with decompensated cirrhosis					
Yes	4	0.94 (0.78–1.14)	0.0	0.70	0.65
No	8	0.83 (0.65–1.05)	70.2	<0.01	
Mean or median follow-up length, year					
≥ 4	7	1.01 (0.88–1.17)	0.0	0.74	<0.01
< 4	5	0.68 (0.54–0.86)	48.2	0.10	

HR, hazard ratio; CI, confidence interval.

^¶^ All subgroup analyses were based on multivariable-adjusted risk estimates.

^†^ *P* for heterogeneity.

^‡^ *P* for interaction between subgroups with meta-regression.

Ignoring a single study in turn did not materially alter the similarity of TDF to entecavir in reducing the risk of HCC (Supplementary Figure 2). Similarly, the initial results remained when we applied various eligibility criteria (Supplementary Table 5).

### Publication bias

We did not find evidence of publication bias with Begg’s test and Egger’s test (all *P*>0.05) and by inspection of funnel plot (Supplementary Figures 3, 4).

## DISCUSSION

Clarifying whether TDF is superior to entecavir for improving the prognosis of CHB patients is of much importance and interest. In the present study, we compared HCC incidence of 80202 CHB patients after initiation of treatment with TDF or entecavir. Our multivariable and propensity-score-matched analyses both showed that TDF was similar to entecavir with respect to the reduction of HCC incidence. In addition, our subgroup analysis further showed that the similarity of TDF to entecavir in reducing HCC incidence was modified by follow-up length.

The majority of previous meta-analyses found that TDF was associated with a lower risk of HCC than entecavir [20–28], which is inconsistent with our study. For example, a recent meta-analysis by Liu et al., including seven studies and 35785 CHB patients, found that patients on TDF treatment were at a lower risk of HCC than those on entecavir treatment (HR 0.75, 95% CI 0.56–0.96) [20]. The publication of several cohort studies motivates us to re-evaluate the potential differences in HCC incidence between TDF and entecavir groups, given that they consistently showed no difference in risk reduction of HCC between two groups [31–33]. Compared with previous meta-analyses, our meta-analysis has several advantages. First, our meta-analysis included the latest studies in this field [31–33], resulting in that our results represent the most up-to-date evidence on this topic. Thus, our results can better reflect the effectiveness of TDF over entecavir in reducing the risk of HCC. Second, our meta-analysis only considered studies providing risk estimates from multivariable or propensity score matching analyses, resulting in that our results are less susceptible to confounders. For example, we excluded a follow-up study by Tsai et al. [34], as it failed to provide the adjusted risk estimate. However, this follow-up study [34] was included in the previous meta-analyses. Third, our meta-analysis conducted predefined subgroup analyses to identify the potential effect modifiers, and showed that the follow-up length was a key effect modifier for the effectiveness of TDF vs entecavir in the prevention of HCC.

It is well established that cirrhosis is a strong risk factor of HCC [35]. Interestingly, a multicenter cohort study found that the annual incidence of HCC differed significantly within and beyond the first 5 years of TDF or entecavir treatment in patients with cirrhosis but not in those without [9], indicating a possible interaction between TDF or entecavir treatment and cirrhosis.

Indeed, it has been found that long-term TDF therapy can result in the regression of cirrhosis in CHB patients [36]. Thus, there is a strong interest in determining whether the observed similarity of TDF to entecavir in HCC prevention could be modified by cirrhosis. To clarify this important issue, we first conducted a subgroup analysis stratified by the presence of cirrhosis. However, we did not observe the expected interaction between TDF or entecavir therapy and cirrhosis (*P*_interaction=_0.09). To verify this observation, we then conducted a subgroup analysis after stratifying for the exclusion of decompensated cirrhosis. Interestingly, the similarity of TDF to entecavir in HCC prevention persisted in two subgroups (*P*_interaction=_0.65). Collectively, these results indicate that the similarity of TDF to entecavir in risk reduction of HCC could not be modified by cirrhosis. However, it should be reminded that our subgroup analysis only included a small number of studies, which raises a possibility that the absence of significant interaction may result from the insufficient power. Hence, more studies with a large sample size are needed to clarify this issue.

Given the widespread use of TDF and entecavir worldwide and the poor prognosis of HCC, our findings have important implications for clinical practice. Current practice guidelines consistently recommend TDF and entecavir as first-line therapies for CHB, without any preference [4–6]. Obviously, the similarity of TDF to entecavir for HCC prevention we observed supports this recommendation. However, TDF has been associated with higher risks of renal impairment [37, 38] and hip fracture [39] compared with entecavir. Hence, when choosing an optimal treatment strategy for a given CHB patient, physicians should consider not only the effectiveness but also the potential comorbidities.

In this study, we observed moderate heterogeneity when evaluating the comparative effectiveness of TDF vs entecavir in reducing HCC incidence. Our subgroup analysis suggests that the difference in follow-up length between included studies could explain the observed heterogeneity. Specifically, the observed similarity of TDF to entecavir for HCC prevention persisted in studies with follow-up length of ≥ 4 years but not in those with follow-up length of < 4 years. It is well established that short-term studies are more subject to reverse causation than long-term studies, which indirectly reminds us that the superiority of TDF over entecavir in reducing the risk of HCC observed in previous meta-analyses may result from this bias. In addition, our sensitivity analysis showed that the observed heterogeneity reduced significantly after excluding studies with sample size of > 10000 [14, 15], suggesting that the difference in sample size between included studies may also explain the observed heterogeneity. Generally, compared with small studies, large studies can document more outcome events of interest, and are performed with more methodological rigor [40].

Our study has several limitations. First, although we extracted risk estimates from multivariable and propensity-score-matched analyses, but we cannot exclude the possibility that our combined results were biased by residual confounding. Second, although not suggested by Begg’s test, Egger’s test. and funnel plot, our combined results might be still influenced by publication bias, as these tests have limited power when there are limited studies. Third, our findings mainly derived from Korean population, and thus might not be generalized to other populations. Finally, we observed moderate heterogeneity for the combined results on the similarity of TDF to entecavir in risk reduction of HCC. Nonetheless, we had identified the sources of heterogeneity through subgroup and sensitivity analyses. Moreover, methodological and clinical heterogeneity exist for all meta-analyses, especially meta-analysis of observational studies.

In conclusion, TDF is similar to entecavir in reducing HCC incidence among treatment-naïve patients with CHB. These findings support the current guidelines that both TDF and entecavir should be considered as first-line agents for CHB treatment. Heterogeneous results of included studies may result from their disparity in follow-up length. Given the inherent limitations of observational data and a small number of included studies, our findings should be treated with caution and need to be validated by future studies.

## MATERIALS AND METHODS

The results of the present study were reported following the Preferred Reporting Items for Systematic Reviews and Meta-analyses (PRISMA) statement [41].

### Search strategy

We conducted an electronic search of PubMed, Embase, Cochrane Library, Web of Science, Wang Fang database, and China National Knowledge Infrastructure database from their inception to November 24, 2020 to identify potentially eligible studies, without any restriction. Supplementary Table 6 shows search strategies used in PubMed and Embase databases in detail. Furthermore, we manually checked the reference lists of pertinent articles to identify additional studies. We did not attempt to contact the original authors to obtain extra information.

### Study selection

All cohort studies comparing the effectiveness of TDF (300 mg/day) and entecavir (0.5 mg/day) in reducing the risk of HCC in treatment-naïve patients with CHB were included if they reported the multivariable-adjusted or propensity-score-matched risk estimates and 95% confidence intervals (CIs). We excluded studies whose study population included treatment-experienced patients or those coinfected with human immunodeficiency virus or hepatitis C virus. We did not consider conference abstract, as its results may change between submitting a meeting abstract and finalizing a manuscript. Based on the prespecified eligibility criteria, two investigators first read titles and abstracts carefully to exclude obviously irrelevant studies, and then scrutinized the full-text to further exclude ineligible studies. Notably, to obtain reliable results, we repeated the process of literature screening several times. Any discrepancies were handled by discussion.

### Data extraction

One investigator extracted the required data with an electronic spreadsheet, and then another investigator checked the data for accuracy. Any discrepancies were settled by discussion. The following data were extracted: first author’s last name, study location, study source, study design, study setting, publication year, sample size, follow-up length, mean age, proportions of males, numbers of cirrhotic patients and HBeAg-positive patients in TDF and entecavir groups, HBV DNA levels, the dose of TDF and entecavir used, the information on the exclusion of patients with decompensated cirrhosis, risk estimates and 95% CIs from multivariable and propensity-score-matched analyses as well as univariable analyses, adjustment variables, and variables used for propensity score matching.

### Quality assessment

Two investigators independently assessed the quality of included studies with the Newcastle-Ottawa quality assessment scale [42]. This scale consists of eight items, which are fallen into three domains (i.e., selection, comparability, and outcome). An individual study could be scored a maximum of nine stars after assessing its three domains. In this meta-analysis, high-quality studies were defined as those earning seven or more stars. Any discrepancies were resolved by discussion.

### Statistical analysis

A random-effects model was used to pool risk estimate from each individual study. Hazard ratio (HR) was used to evaluate the difference in HCC incidence between TDF and entecavir groups. Sub-distribution hazard ratio was directly regarded as equivalent to HR [12, 14]. The Hedges Q statistic (a *P* < 0.10 suggesting statistical significance) and the *I*^2^ statistic (an *I*^2^ of < 50%, 50.0%-75.0%, and > 75.0% representing low, moderate, and substantial heterogeneity, respectively) were used to qualitatively and quantitatively reflect the between-study heterogeneity, respectively.

As confounding bias is always a major concern in observational studies, we used the following strategies to control and reflect the potential effects of confounders on outcomes of interest: we first pooled risk estimates from multivariable analyses to obtain our primary data that quantified the effectiveness of TDF vs entecavir in HCC prevention; we then pooled risk estimates from propensity-score-matched analyses to minimize the confounding effect caused by the differences in baseline characteristics; finally, we pooled unadjusted risk estimates for comparison with the results of multivariable and propensity-score-matched analyses.

To identify the potential effect modifiers, we conducted a series of predefined subgroup analyses after stratifying for study location (Korea vs no-Korea), study source (population-based vs hospital-based), study setting (multicenter vs single-center), cirrhosis (yes vs no), exclusion of patients with decompensated cirrhosis (yes vs no), and follow-up length (≥ 4 vs < 4 years). A *P*_interaction_ for the difference between subgroups was calculated via meta-regression. To determine the robustness of pooled results, we conducted the following sensitivity analyses: omitting a single study in turn and using various eligibility criteria. We used Begg’s test [43], Egger’s test [44], and a funnel plot to evaluate publication bias. We conducted all data analyses through STATA software (version12.0, StataCorp, College Station, TX). The results were considered statistically significant when a two-tailed *P* value was less than 0.05.

## Supplementary Material

Supplementary Figures

Supplementary Table 1

Supplementary Table 2

Supplementary Table 3

Supplementary Table 4

Supplementary Tables 5 and 6

## References

[1] Polaris Observatory Collaborators. Global prevalence, treatment, and prevention of hepatitis B virus infection in 2016: a modelling study. Lancet Gastroenterol Hepatol. 2018; 3:383–403. 10.1016/S2468-1253(18)30056-6 29599078

[2] Chen CJ, Yang HI, Su J, Jen CL, You SL, Lu SN, Huang GT, Iloeje UH, and REVEAL-HBV Study Group. Risk of hepatocellular carcinoma across a biological gradient of serum hepatitis B virus DNA level. JAMA. 2006; 295:65–73. 10.1001/jama.295.1.65 16391218

[3] Iloeje UH, Yang HI, Su J, Jen CL, You SL, Chen CJ, and Risk Evaluation of Viral Load Elevation and Associated Liver Disease/Cancer-In HBV (the REVEAL-HBV) Study Group. Predicting cirrhosis risk based on the level of circulating hepatitis B viral load. Gastroenterology. 2006; 130:678–86. 10.1053/j.gastro.2005.11.016 16530509

[4] European Association for the Study of the Liver. Electronic address: easloffice@easloffice.eu; European Association for the Study of the Liver. EASL 2017 Clinical Practice Guidelines on the management of hepatitis B virus infection. J Hepatol. 2017; 67:370–98. 10.1016/j.jhep.2017.03.021 28427875

[5] Terrault NA, Lok AS, McMahon BJ, Chang KM, Hwang JP, Jonas MM, Brown RS Jr, Bzowej NH, Wong JB. Update on prevention, diagnosis, and treatment of chronic hepatitis B: AASLD 2018 hepatitis B guidance. Hepatology. 2018; 67:1560–99. 10.1002/hep.29800 29405329 PMC5975958

[6] Sarin SK, Kumar M, Lau GK, Abbas Z, Chan HL, Chen CJ, Chen DS, Chen HL, Chen PJ, Chien RN, Dokmeci AK, Gane E, Hou JL, et al. Asian-pacific clinical practice guidelines on the management of hepatitis B: a 2015 update. Hepatol Int. 2016; 10:1–98. 10.1007/s12072-015-9675-4 26563120 PMC4722087

[7] Liu K, Choi J, Le A, Yip TC, Wong VW, Chan SL, Chan HL, Nguyen MH, Lim YS, Wong GL. Tenofovir disoproxil fumarate reduces hepatocellular carcinoma, decompensation and death in chronic hepatitis B patients with cirrhosis. Aliment Pharmacol Ther. 2019; 50:1037–48. 10.1111/apt.15499 31524304

[8] Ko KL, To WP, Mak LY, Seto WK, Ning Q, Fung J, Lai CL, Yuen MF. A large real-world cohort study examining the effects of long-term entecavir on hepatocellular carcinoma and HBsAg seroclearance. J Viral Hepat. 2020; 27:397–406. 10.1111/jvh.13237 31755196

[9] Papatheodoridis GV, Idilman R, Dalekos GN, Buti M, Chi H, van Boemmel F, Calleja JL, Sypsa V, Goulis J, Manolakopoulos S, Loglio A, Siakavellas S, Keskın O, et al. The risk of hepatocellular carcinoma decreases after the first 5 years of entecavir or tenofovir in caucasians with chronic hepatitis B. Hepatology. 2017; 66:1444–53. 10.1002/hep.29320 28622419

[10] Lee SW, Kwon JH, Lee HL, Yoo SH, Nam HC, Sung PS, Nam SW, Bae SH, Choi JY, Yoon SK, Han NI, Jang JW. Comparison of tenofovir and entecavir on the risk of hepatocellular carcinoma and mortality in treatment-naïve patients with chronic hepatitis B in Korea: a large-scale, propensity score analysis. Gut. 2020; 69:1301–08. 10.1136/gutjnl-2019-318947 31672838 PMC7306978

[11] Kim SU, Seo YS, Lee HA, Kim MN, Lee YR, Lee HW, Park JY, Kim DY, Ahn SH, Han KH, Hwang SG, Rim KS, Um SH, et al. A multicenter study of entecavir vs. Tenofovir on prognosis of treatment-naïve chronic hepatitis B in South Korea. J Hepatol. 2019; 71:456–64. 10.1016/j.jhep.2019.03.028 30959156

[12] Hsu YC, Wong GL, Chen CH, Peng CY, Yeh ML, Cheung KS, Toyoda H, Huang CF, Trinh H, Xie Q, Enomoto M, Liu L, Yasuda S. Tenofovir versus entecavir for hepatocellular carcinoma prevention in an international consortium of chronic hepatitis B. Am J Gastroenterol. 2020; 115:271–80. 10.14309/ajg.0000000000000428 31634265

[13] Kim BG, Park NH, Lee SB, Lee H, Lee BU, Park JH, Jung SW, Jeong ID, Bang SJ, Shin JW. Mortality, liver transplantation and hepatic complications in patients with treatment-naïve chronic hepatitis B treated with entecavir vs tenofovir. J Viral Hepat. 2018; 25:1565–75. 10.1111/jvh.12971 29998592

[14] Yip TC, Wong VW, Chan HL, Tse YK, Lui GC, Wong GL. Tenofovir is associated with lower risk of hepatocellular carcinoma than entecavir in patients with chronic HBV infection in China. Gastroenterology. 2020; 158:215–25.e6. 10.1053/j.gastro.2019.09.025 31574268

[15] Choi J, Kim HJ, Lee J, Cho S, Ko MJ, Lim YS. Risk of Hepatocellular Carcinoma in Patients Treated With Entecavir vs Tenofovir for Chronic Hepatitis B: A Korean Nationwide Cohort Study. JAMA Oncol. 2019; 5:30–36. 10.1001/jamaoncol.2018.4070 30267080 PMC6439769

[16] Ha I, Chung JW, Jang ES, Jeong SH, Kim JW. Comparison of the on-treatment risks for hepatocellular carcinoma between entecavir and tenofovir: a propensity score matching analysis. J Gastroenterol Hepatol. 2020; 35:1774–81. 10.1111/jgh.15031 32154938

[17] Oh H, Yoon EL, Jun DW, Ahn SB, Lee HY, Jeong JY, Kim HS, Jeong SW, Kim SE, Shim JJ, Sohn JH, Cho YK, and Long-Term Safety of Entecavir and Tenofovir in Patients With Treatment-Naive Chronic Hepatitis B Virus (CHB) Infection (SAINT) Study. No difference in incidence of hepatocellular carcinoma in patients with chronic hepatitis B virus infection treated with entecavir vs tenofovir. Clin Gastroenterol Hepatol. 2020; 18:2793–802.e6. 10.1016/j.cgh.2020.02.046 32135246

[18] Papatheodoridis GV, Dalekos GN, Idilman R, Sypsa V, Van Boemmel F, Buti M, Calleja JL, Goulis J, Manolakopoulos S, Loglio A, Papatheodoridi M, Gatselis N, Veelken R, et al. Similar risk of hepatocellular carcinoma during long-term entecavir or tenofovir therapy in caucasian patients with chronic hepatitis B. J Hepatol. 2020; 73:1037–45. 10.1016/j.jhep.2020.06.011 32553667

[19] Su F, Berry K, Ioannou GN. No difference in hepatocellular carcinoma risk between chronic hepatitis B patients treated with entecavir versus tenofovir. Gut. 2021; 70:370–78. 10.1136/gutjnl-2019-319867 32229544

[20] Liu H, Shi Y, Hayden JC, Ryan PM, Rahmani J, Yu G. Tenofovir treatment has lower risk of hepatocellular carcinoma than entecavir treatment in patients with chronic hepatitis B: a systematic review and meta-analysis. Liver Cancer. 2020; 9:468–76. 10.1159/000507253 32999872 PMC7506291

[21] Teng YX, Li MJ, Xiang BD, Zhong JH. Tenofovir may be superior to entecavir for preventing hepatocellular carcinoma and mortality in individuals chronically infected with HBV: a meta-analysis. Gut. 2020; 69:1900–02. 10.1136/gutjnl-2019-320326 31843789

[22] Zhang Z, Zhou Y, Yang J, Hu K, Huang Y. The effectiveness of TDF versus ETV on incidence of HCC in CHB patients: a meta analysis. BMC Cancer. 2019; 19:511. 10.1186/s12885-019-5735-9 31142283 PMC6542001

[23] Choi WM, Choi J, Lim YS. Effects of tenofovir vs entecavir on risk of hepatocellular carcinoma in patients with chronic HBV infection: a systematic review and meta-analysis. Clin Gastroenterol Hepatol. 2021; 2:246–58.e9. 10.1016/j.cgh.2020.05.008 32407970

[24] Dave S, Park S, Murad MH, Barnard A, Prokop L, Adams LA, Singh S, Loomba R. Comparative Effectiveness of Entecavir Versus Tenofovir for Preventing Hepatocellular Carcinoma in Patients with Chronic Hepatitis B: A Systematic Review and Meta-Analysis. Hepatology. 2020. [Epub ahead of print]. 10.1002/hep.31267 32277491 PMC8022893

[25] Gu L, Yao Q, Shen Z, He Y, Ng DM, Yang T, Chen B, Chen P, Mao F, Yu Q. Comparison of tenofovir versus entecavir on reducing incidence of hepatocellular carcinoma in chronic hepatitis B patients: a systematic review and meta-analysis. J Gastroenterol Hepatol. 2020; 35:1467–76. 10.1111/jgh.15036 32180249

[26] Li M, Lv T, Wu S, Wei W, Wu X, Ou X, Ma H, Chow SC, Kong Y, You H, Jia J. Tenofovir versus entecavir in lowering the risk of hepatocellular carcinoma development in patients with chronic hepatitis B: a critical systematic review and meta-analysis. Hepatol Int. 2020; 14:105–14. 10.1007/s12072-019-10005-0 31898210

[27] Yuan BH, Zhu YK, Li RH. Letter: is tenofovir superior to entecavir for hepatocellular carcinoma prevention in chronic hepatitis B? Aliment Pharmacol Ther. 2020; 51:314–15. 10.1111/apt.15603 31880015

[28] Cheung KS, Mak LY, Liu SH, Cheng HM, Seto WK, Yuen MF, Lai CL. Entecavir vs tenofovir in hepatocellular carcinoma prevention in chronic hepatitis B infection: a systematic review and meta-analysis. Clin Transl Gastroenterol. 2020; 11:e00236. 10.14309/ctg.0000000000000236 33031195 PMC7544163

[29] Tseng CH, Hsu YC, Chen TH, Ji F, Chen IS, Tsai YN, Hai H, Thuy LT, Hosaka T, Sezaki H, Borghi JA, Cheung R, Enomoto M, Nguyen MH. Hepatocellular carcinoma incidence with tenofovir versus entecavir in chronic hepatitis B: a systematic review and meta-analysis. Lancet Gastroenterol Hepatol. 2020; 5:1039–52. 10.1016/S2468-1253(20)30249-1 33007228

[30] Wang X, Liu X, Dang Z, Yu L, Jiang Y, Wang X, Yan Z. Nucleos(t)ide analogues for reducing hepatocellular carcinoma in chronic hepatitis B patients: a systematic review and meta-analysis. Gut Liver. 2020; 14:232–47. 10.5009/gnl18546 31158948 PMC7096226

[31] Shin JW, Jeong J, Jung SW, Lee SB, Park BR, Kim MJ, Park EJ, Park NH. Comparable incidence of hepatocellular carcinoma in chronic hepatitis B patients treated with entecavir or tenofovir. Dig Dis Sci. 2020. [Epub ahead of print]. 10.1007/s10620-020-06375-3 32524416

[32] Hu TH, Yueh-Hsia Chiu S, Tseng PL, Chen CH, Lu SN, Wang JH, Hung CH, Kee KM, Lin MT, Chang KC, Lin MC, Chien RN. Five-year comparative risk of hepatocellular carcinoma development under entecavir or tenofovir treatment-naïve patients with chronic hepatitis B-related compensated cirrhosis in Taiwan. Aliment Pharmacol Ther. 2020; 52:1695–706. 10.1111/apt.16116 33111400

[33] Ha Y, Chon YE, Kim MN, Lee JH, Hwang SG. Hepatocellular carcinoma and death and transplantation in chronic hepatitis B treated with entecavir or tenofovir disoproxil fumarate. Sci Rep. 2020; 10:13537. 10.1038/s41598-020-70433-z 32782369 PMC7419516

[34] Tsai MC, Chen CH, Hu TH, Lu SN, Lee CM, Wang JH, Hung CH. Long-term outcomes of hepatitis B virus-related cirrhosis treated with nucleos(t)ide analogs. J Formos Med Assoc. 2017; 116:512–21. 10.1016/j.jfma.2016.08.006 27720344

[35] Persson EC, Quraishi SM, Welzel TM, Carreon JD, Gridley G, Graubard BI, McGlynn KA. Risk of liver cancer among US male veterans with cirrhosis, 1969-1996. Br J Cancer. 2012; 107:195–200. 10.1038/bjc.2012.193 22588556 PMC3389404

[36] Marcellin P, Gane E, Buti M, Afdhal N, Sievert W, Jacobson IM, Washington MK, Germanidis G, Flaherty JF, Aguilar Schall R, Bornstein JD, Kitrinos KM, Subramanian GM, et al. Regression of cirrhosis during treatment with tenofovir disoproxil fumarate for chronic hepatitis B: a 5-year open-label follow-up study. Lancet. 2013; 381:468–75. 10.1016/S0140-6736(12)61425-1 23234725

[37] Udompap P, Kim D, Ahmed A, Kim WR. Longitudinal trends in renal function in chronic hepatitis B patients receiving oral antiviral treatment. Aliment Pharmacol Ther. 2018; 48:1282–89. 10.1111/apt.15020 30370967

[38] Wong GL, Chan HL, Tse YK, Yip TC, Lam KL, Lui GC, Szeto CC, Wong VW. Chronic kidney disease progression in patients with chronic hepatitis B on tenofovir, entecavir, or no treatment. Aliment Pharmacol Ther. 2018; 48:984–92. 10.1111/apt.14945 30125952

[39] Wong GL, Tse YK, Wong VW, Yip TC, Tsoi KK, Chan HL. Long-term safety of oral nucleos(t)ide analogs for patients with chronic hepatitis B: a cohort study of 53,500 subjects. Hepatology. 2015; 62:684–93. 10.1002/hep.27894 25973979

[40] Nnoaham KE, Webster P, Kumbang J, Kennedy SH, Zondervan KT. Is early age at menarche a risk factor for endometriosis? a systematic review and meta-analysis of case-control studies. Fertil Steril. 2012; 98:702–12.e6. 10.1016/j.fertnstert.2012.05.035 22728052 PMC3502866

[41] Stroup DF, Berlin JA, Morton SC, Olkin I, Williamson GD, Rennie D, Moher D, Becker BJ, Sipe TA, Thacker SB. Meta-analysis of observational studies in epidemiology: a proposal for reporting. Meta-analysis Of Observational Studies in Epidemiology (MOOSE) group. JAMA. 2000; 283:2008–12. 10.1001/jama.283.15.2008 10789670

[42] Wells G, Shea B, O’Connell D, Peterson J, Welch V, Losos M, Tugwell P. The Newcastle-Ottawa Scale (NOS) for assessing the quality of nonrandomised studies in meta-analyses. 2000.

[43] Begg CB, Mazumdar M. Operating characteristics of a rank correlation test for publication bias. Biometrics. 1994; 50:1088–101. 10.2307/2533446 7786990

[44] Egger M, Davey Smith G, Schneider M, Minder C. Bias in meta-analysis detected by a simple, graphical test. BMJ. 1997; 315:629–34. 10.1136/bmj.315.7109.629 9310563 PMC2127453

