# Supplementary material for: No difference in hepatocellular carcinoma risk in chronic hepatitis B patients treated with tenofovir vs entecavir: evidence from an updated meta-analysis
Source: Aging (Albany NY). 2021 Feb 26;13(5):7147–65. doi: 10.18632/aging.202573 (PMC7993671; doi:10.18632/aging.202573)
Supplement: Supplementary Figures [file aging-13-202573-s001.pdf]

## SUPPLEMENTARY FIGURES

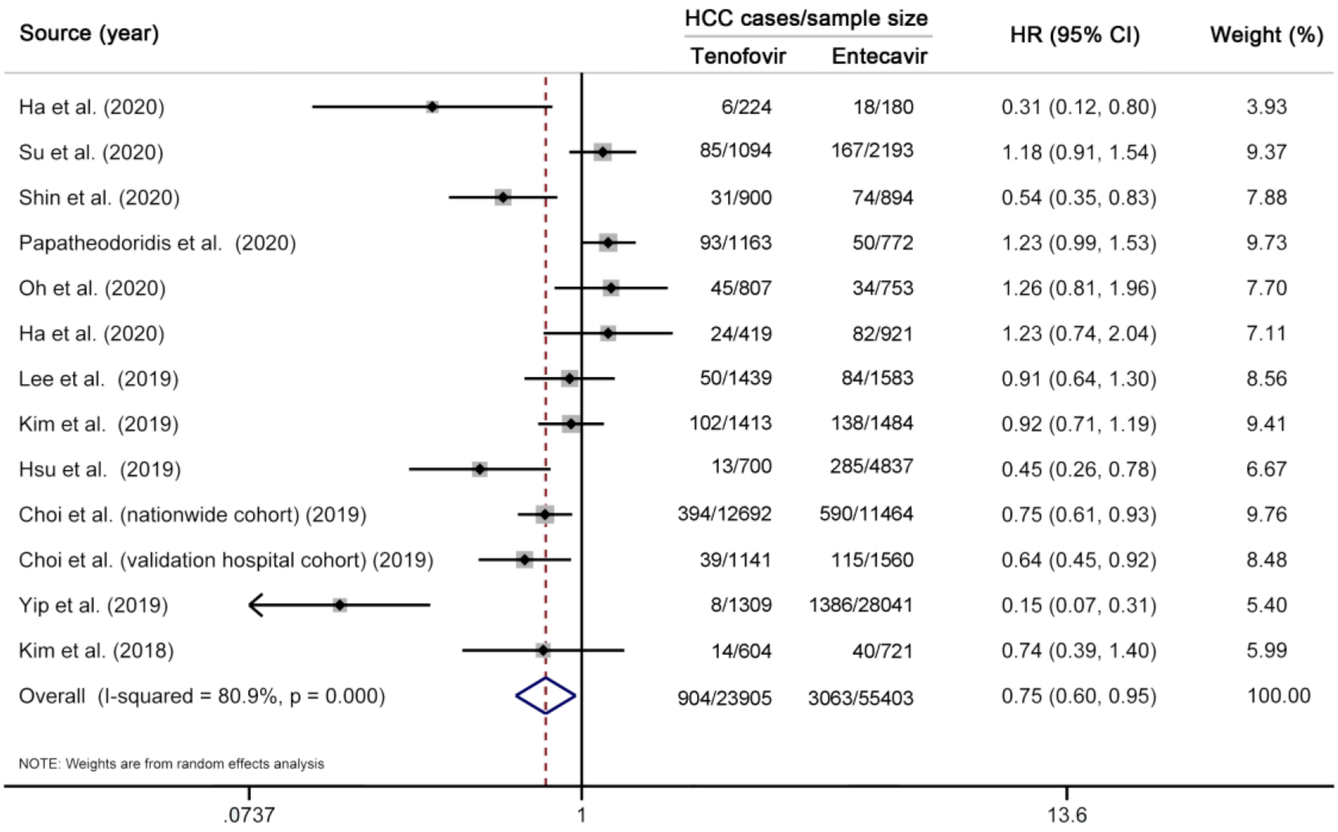


**Supplementary Figure 1. Meta-analysis comparing the effectiveness of TDF vs entecavir in reducing HCC risk.** Note that this analysis is based on unadjusted risk estimates. The squares represent risk estimate of each included study, with the area reflecting the weight assigned to the study. The horizontal line across each square represents 95% CI. The diamond represents the pooled risk estimate, with width representing 95% CI. TDF, tenofovir disoproxil fumarate; HCC, hepatocellular carcinoma; HR, hazard ratio; CI, confidence interval.

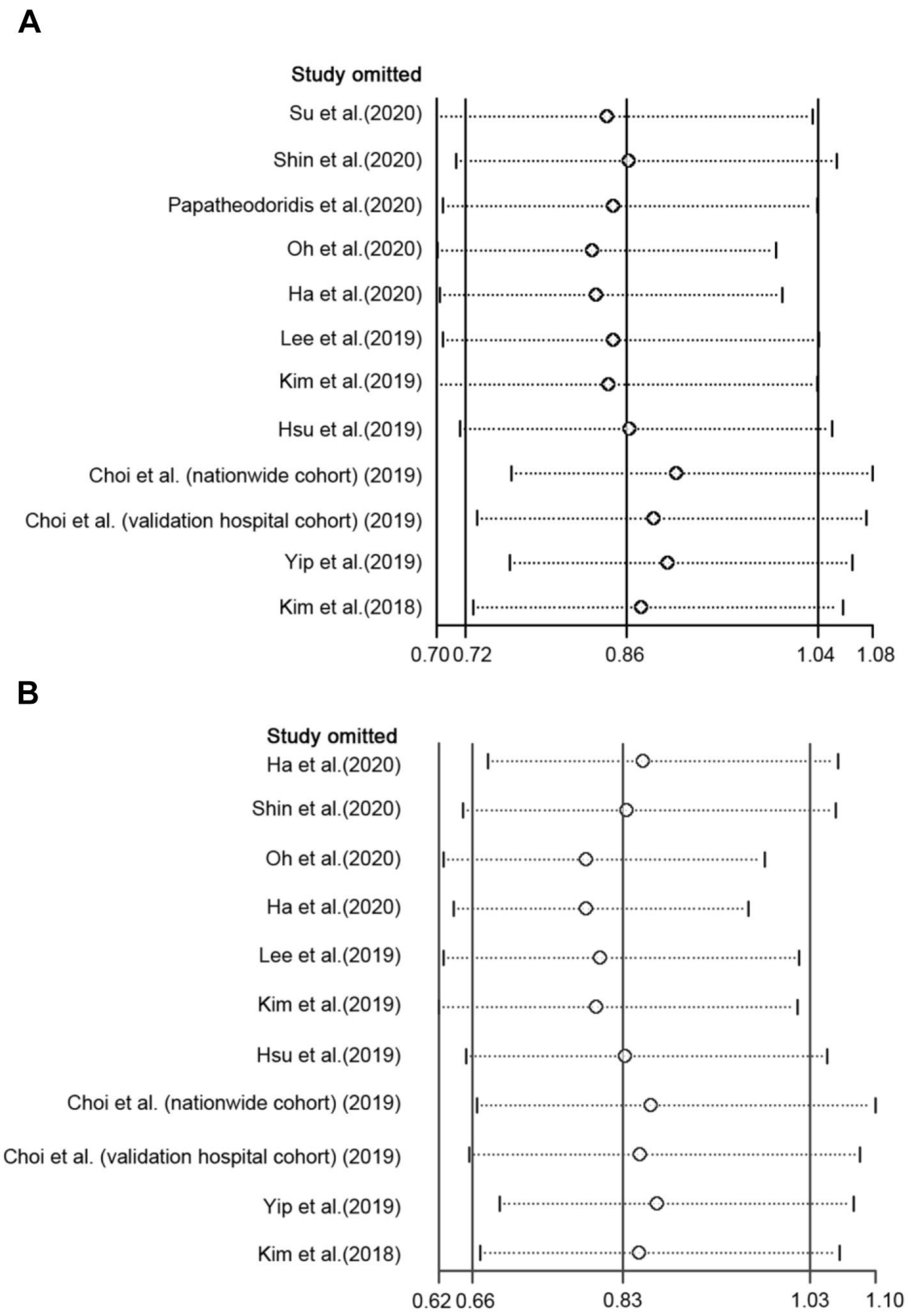


**Supplementary Figure 2. Sensitivity analyses on the effectiveness of TDF vs entecavir in reducing HCC risk: the exclusion of a single study in turn**. (**A**) sensitivity analysis based on multivariable-adjusted risk estimates. (**B**) sensitivity analysis based on propensity-score-matched risk estimates. The study cited on the left is the one left out in each turn. The solid circle represents the summary risk estimates after exclusion of a single study, and the corresponding dot line represents 95% confidence interval. The middle vertical solid line represents summary risk estimates of all included studies, and left and right vertical solid line represent lower limit and upper limit, respectively.

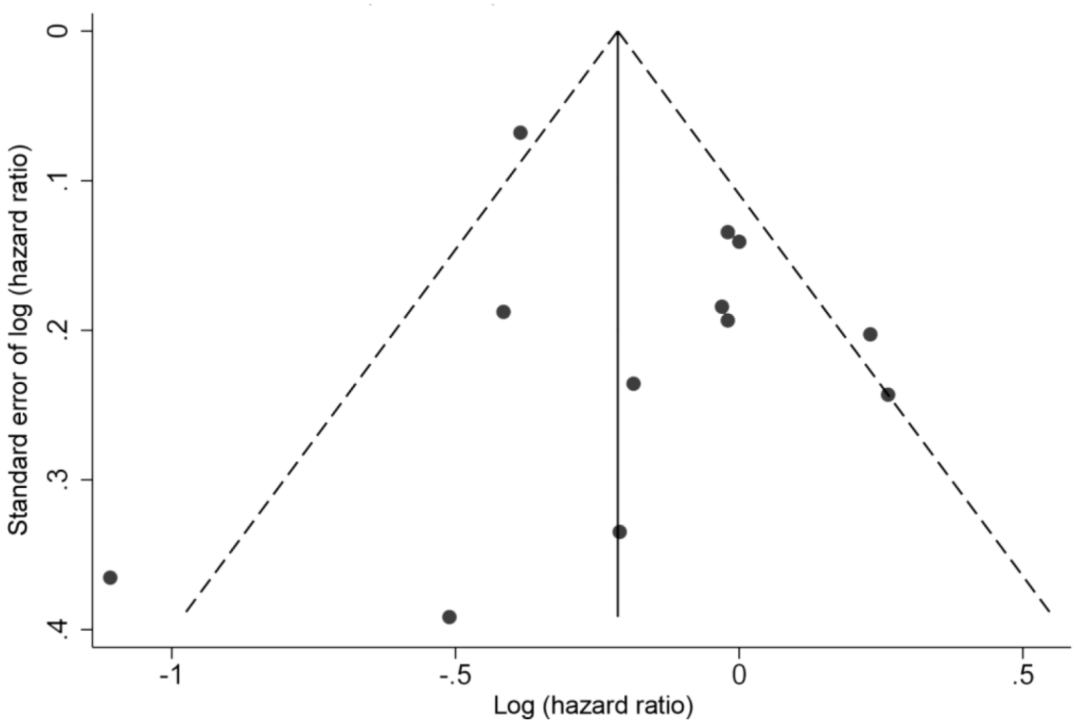


**Supplementary Figure 3. The funnel plot of included studies**. Note that this analysis is based on multivariable-adjusted risk estimates. The funnel plot is a graphical representation of the logarithm of study-specific hazard ratio vs standard error of the logarithm of study-specific hazard ratio. The dotted lines represent pseudo 95% confidence intervals.

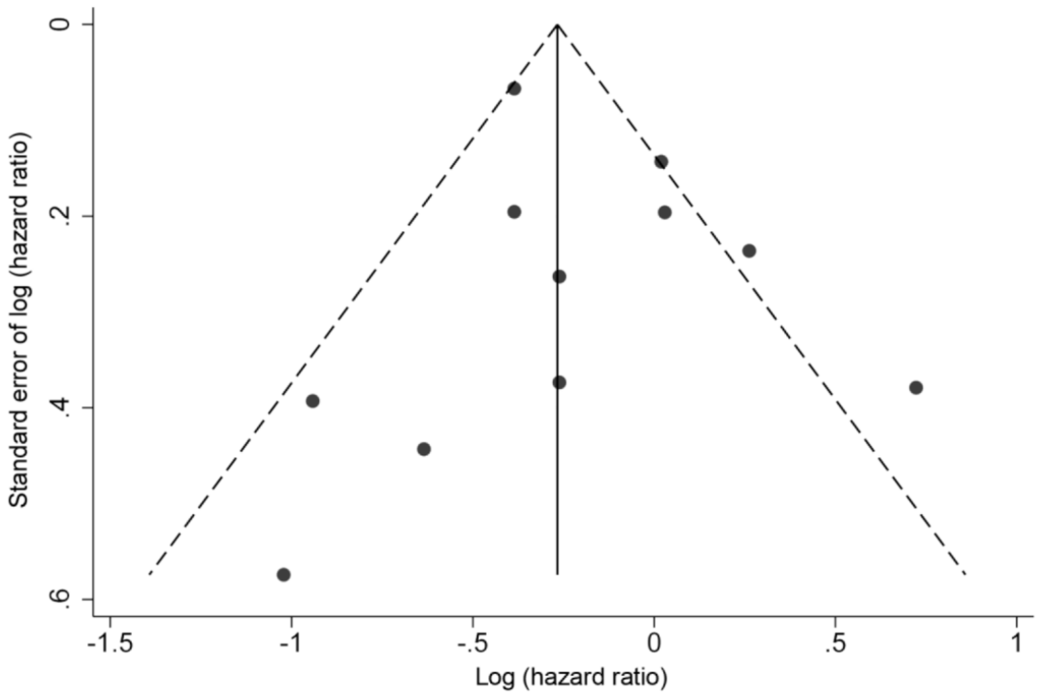


**Supplementary Figure 4. The funnel plot of included studies.** Note that this analysis is based on propensity-score-matched risk estimates. The funnel plot is a graphical representation of the logarithm of study-specific hazard ratio vs standard error of the logarithm of study-specific hazard ratio. The dotted lines represent pseudo 95% confidence intervals.
