# Supplementary material for: No difference in hepatocellular carcinoma risk in chronic hepatitis B patients treated with tenofovir vs entecavir: evidence from an updated meta-analysis
Source: Aging (Albany NY). 2021 Feb 26;13(5):7147–65. doi: 10.18632/aging.202573 (PMC7993671; doi:10.18632/aging.202573)
Supplement: Supplementary Table 1 [file aging-13-202573-s002.pdf]

SUPPLEMENTARY TABLE

Supplementary Table 1. Excluded studies after checking the full text and primary reason for exclusion.

Author	Year	Study description	Primary reason for exclusion
Pol et al. [1]	2019	This prospective study found that the risk of liver-related events was not different between tenofovir and entecavir group. However, the study was published in the form of conference abstract.	Conference abstract
Lee et al. [2]	2019	This propensity score analysis compared the effect of tenofovir and entecavir on the risk of hepatocellular carcinoma and liver-related events in patients with CHB. It is noteworthy that this study is published in the form of conference abstract and is a duplicate report of an included study [3].	Duplicate report of included study
Le et al. [4]	2019	This is a multicenter retrospective cohort study of CHB patients; its primary purpose was to evaluate the long-term safety and efficacy of tenofovir and entecavir. This study reported the number of patients diagnosed with liver cancer in tenofovir and entecavir groups but not adjusted risk estimates.	Adjusted risk estimates were not available
Kim et al. [5]	2019	This study is a retrospective cohort study, and found that treatment with tenofovir was associated with a reduced risk of HCC compared with treatment with entecavir. However, the study was published in the form of conference abstract.	Conference abstract
Gordon et al. [6]	2019	This prospective cohort study suggested that the risk of HCC in patients treated with tenofovir versus entecavir might vary by race group. However, the study was published in the form of conference abstract.	Conference abstract
Lee et al. [7]	2018	This longitudinal observational analysis compared the risk of developing HCC in treatment-naïve CHB patients and provided the relevant hazard ratio. Of note, this study is published in the form of conference abstract and is a duplicate report of an included study [8].	Duplicate report of included study
Kim et al. [9]	2018	This retrospective study reported the annual incidence of HCC in tenofovir and entecavir groups (0.85% versus 1.27%), with 3 cases in tenofovir group (3/112, 2.7%) and 13 in entecavir group (13/191, 6.8%). However, it failed to provide the relevant adjusted risk estimates.	Adjusted risk estimates were not available
Ha et al. [10]	2018	This study is a retrospective cohort study, and found that there was no difference in risk reduction of HCC between tenofovir and entecavir. However, the study was published in the form of conference abstract. Also, this study is a duplicate report of an included study [11].	Conference abstract
Tsai et al. [12]	2017	This follow-up study documented a total of 56 HCC cases in a cohort of 546 CHB patients with cirrhosis on nucleos(t)ide analog therapy. The authors did not report the relevant adjusted risk estimates of developing HCC.	Adjusted risk estimates were not available
Riveiro-Barciela et al. [13]	2017	This study aimed to assess the effectiveness and safety of tenofovir or entecavir in CHB patients. The authors provided the number of HCC cases in tenofovir and entecavir groups (11 in tenofovir group and 3 in entecavir group) but not the corresponding adjusted risk estimates.	Adjusted risk estimates were not available
Papatheodoridis et al. [14]	2017	This is a multicenter cohort study involving 1951 CHB patients. The primary aim of the study was to determine the HCC incidence in patients receiving tenofovir or entecavir treatment. The authors only reported the overall HCC incidence in their study population.	Overall HCC incidence in the whole study population.
Choi et al. [15]	2017	This cohort study found that tenofovir treatment conferred a reduced risk of HCC but a similar risk death or transplantation compared with entecavir treatment. However, the study was published in the form of conference abstract.	Conference abstract
Kramer et al. [16]	2015	This study examined the effect of tenofovir versus entecavir on the risk of HCC in CHB patients. However, the authors did not exclude patients with HIV/HCV infection.	Including patients with HIV/HCV infection.
Idilman et al. [17]	2015	This study documented a total of 17 HCC cases in a cohort of 355 CHB patients and showed that there was no significant difference in HCC incidence between tenofovir and entecavir groups. Importantly, the authors did not provide the relevant adjusted risk estimates of developing HCC.	Adjusted risk estimates were not available.
Goyal et al. [18]	2015	This study aimed to evaluate the efficacy and outcome of CHB patients receiving	Adjusted risk estimates

		tenofovir and entecavir treatment. The study showed that 6 patients in tenofovir group and 4 patients in entecavir group developed HCC during follow up. Note that the authors did not provide the relevant adjusted risk estimates.	were not available.
Hsu et al. [19]	2014	This study included a total of 210 CHB patients receiving antiviral treatment (lamivudine, telbivudine, entecavir, and tenofovir). During a median follow-up of 25.2 months, the authors observed 35 HCC cases (1 in lamivudine group, 2 in telbivudine group, and 32 in entecavir group). It is noteworthy that the authors did not provide the relevant adjusted risk estimates.	Adjusted risk estimates were not available.
Hanumantharaya et al. [20]	2014	This study included a total of 132 CHB patients on antiviral treatment (84 receiving tenofovir and 48 receiving entecavir). The authors only reported the number of HCC cases in tenofovir and entecavir groups (2 patients in tenofovir group and 2 patients in entecavir group) but not the relevant adjusted risk estimates.	Adjusted risk estimates were not available.
Coffin et al. [21]	2014	The study aimed to determine the HCC incidence of HCC in CHB patients receiving nucleos(t)ide analogues treatment. The study documented a total of 11 HCC cases over a median follow-up of 3.2 years, with 1 in entecavir group (1/127) and 3 in tenofovir group (3/132). The authors revealed that the annual incidence of HCC in the study cohort was 0.9% per year. However, importantly, the authors did not report the relevant adjusted risk estimates.	Adjusted risk estimates were not available
Koklu et al. [22]	2013	This is a retrospective analysis of 227 CHB patients, with 72 patients receiving tenofovir and 77 patients receiving entecavir. The authors reported the number of HCC cases in tenofovir- and entecavir-treated patients (2 in tenofovir group and 4 in entecavir group). The authors did not provide the relevant adjusted risk estimates.	Adjusted risk estimates were not available

Note that the reference numbers in Supplementary Table 2 refer to the reference list presented below.

REFERENCES

1. Pol，Stanislas. Tenofovir versus entecavir in HBV chronic infection: impact on HCC and other liver-related complications occurrences. Paper presented at: Hepatology. 2019.

2. Lee SW, Kwon JH, Lee HL, Yoo SH, Nam HC, Sung PS, Nam SW, Bae SH, Choi JY, Yoon SK, Han NI, Jang JW. Comparison of tenofovir and entecavir on the risk of hepatocellular carcinoma and liver-related events in patients with chronic hepatitis B: A propensity score analysis. J Hepatol. 2019;70:472.

3. Lee SW, Kwon JH, Lee HL, Yoo SH, Nam HC, Sung PS, Nam SW, Bae SH, Choi JY, Yoon SK, Han NI, Jang JW. Comparison of tenofovir and entecavir on the risk of hepatocellular carcinoma and mortality in treatment-naïve patients with chronic hepatitis B in Korea: a large-scale, propensity score analysis. Gut. 2020; 69:1301–08. https://doi.org/10.1136/gutjnl-2019-318947 PMID:31672838

4. Le S, Ratnam D, Van Leest R, Kemp W, Roberts S, Nicoll A, Gow P, Bell S, Sood S, Arachchi N, Lubel J, Ryan M, Howell J, et al. Real-world efficacy of long-term entecavir and tenofovir in patients with chronic hepatitis B. J Gastroenterol Hepatol. 2019; 34:95.

5. Kim W, Telep L, Lu M, Ramroth H, Flaherty JF, Gaggar A, Chokkalingam A, Gordon SC. Risk of incident hepatocellular carcinoma in hepatitis B-infected patients treated with tenofovir disoproxil fumarate versus entecavir: a US administrative claims analysis. Paper presented at: Hepatology. 2019. https://doi.org/10.1136/gutjnl-2020-IDDF.140

6. Gordon SC, Zhou Y, Li J, Rupp LB, Boscarino JA, Daida YG, Schmidt MA, Trudeau S, Lu M. Effect of treatment of hepatitis B patients with tenofovir disoproxil or entecavir on risk of hepatocellular cancer death in a U.S. Cohort. J Hepatol. 2019; 70:e147. https://doi.org/10.1016/S0618-8278(19)30259-2

7. Lee SB, Bang SJ, Shin JW, Park NH, Lee H, Lee BU, Park JH, Kim BG, Jung SW, Jeong ID. Mortality, liver transplantation, and hepatic complications in patients with treatment-naïve chronic hepatitis B treated with entecavir vs tenofovir. Hepatol Int. 2018;12:S324-S325.

8. Kim BG, Park NH, Lee SB, Lee H, Lee BU, Park JH, Jung SW, Jeong ID, Bang SJ, Shin JW. Mortality, liver transplantation and hepatic complications in patients with treatment-naïve chronic hepatitis B treated with entecavir vs tenofovir. J Viral Hepat. 2018; 25:1565–75. https://doi.org/10.1111/jvh.12971 PMID:29998592

9. Kim YM, Shin HP, Lee JI, Joo KR, Cha JM, Jeon JW, Yoon JY, Kwak MS. Real-world single-center experience with entecavir and tenofovir disoproxil fumarate in treatment-naïve and experienced patients with chronic hepatitis B. Saudi J Gastroenterol. 2018; 24:326–35. https://doi.org/10.4103/sjg.SJG_49_18 PMID:30004042

10. Ha I, Jang ES, Jeong SH, Kim JW. Entecavir and tenofovir have comparable effect on the risk for

hepatocellular carcinoma in chronic hepatitis B: Propensity score matching analysis. Hepatology. 2018; 68:845A.

11. Ha I, Chung JW, Jang ES, Jeong SH, Kim JW. Comparison of the on-treatment risks for hepatocellular carcinoma between entecavir and tenofovir: a propensity score matching analysis. J Gastroenterol Hepatol. 2020; 35:1774–81. https://doi.org/10.1111/jgh.15031 PMID:32154938

12. Tsai MC, Chen CH, Hu TH, Lu SN, Lee CM, Wang JH, Hung CH. Long-term outcomes of hepatitis B virus-related cirrhosis treated with nucleos(t)ide analogs. J Formos Med Assoc. 2017; 116:512–21. https://doi.org/10.1016/j.jfma.2016.08.006 PMID:27720344

13. Riveiro-Barciela M, Tabernero D, Calleja JL, Lens S, Manzano ML, Rodríguez FG, Crespo J, Piqueras B, Pascasio JM, Comas C, Gutierrez ML, Aguirre A, Suárez E, et al. Effectiveness and safety of entecavir or tenofovir in a spanish cohort of chronic hepatitis B patients: validation of the page-B score to predict hepatocellular carcinoma. Dig Dis Sci. 2017; 62:784–93. https://doi.org/10.1007/s10620-017-4448-7 PMID:28078526

14. Papatheodoridis GV, Idilman R, Dalekos GN, Buti M, Chi H, van Boemmel F, Calleja JL, Sypsa V, Goulis J, Manolakopoulos S, Loglio A, Siakavellas S, Keskın O, et al. The risk of hepatocellular carcinoma decreases after the first 5 years of entecavir or tenofovir in caucasians with chronic hepatitis B. Hepatology. 2017; 66:1444–53. https://doi.org/10.1002/hep.29320 PMID:28622419

15. Choi J, Kim HJ, Lee J, Cho S, Ko MJ, Lim YS. Risk of Hepatocellular Carcinoma in Patients Treated With Entecavir vs Tenofovir for Chronic Hepatitis B: A Korean Nationwide Cohort Study. JAMA Oncol. 2019; 5:30–36. https://doi.org/10.1001/jamaoncol.2018.4070 PMID:30267080

16. Kramer JR, Mittal S, Richardson P, El-Serag HB, Kanwal F. Comparative effectiveness of tenofovir vs. Entecavir in reducing the risk of hepatocellular carcinoma in a U.S. Cohort of patients with chronic Hepatitis B virus infection. Hepatology. 2015; 62:335A–336A.

17. Idilman R, Gunsar F, Koruk M, Keskin O, Meral CE, Gulsen M, Elhan AH, Akarca US, Yurdaydin C. Long-term entecavir or tenofovir disoproxil fumarate therapy in treatment-naïve chronic hepatitis B patients in the real-world setting. J Viral Hepat. 2015; 22:504–10. https://doi.org/10.1111/jvh.12358 PMID:25431108

18. Goyal SK, Dixit VK, Shukla SK, Ghosh J, Behera M, Tripathi M, Gupta N, Ranjan A, Jain AK. Prolonged use of tenofovir and entecavir in hepatitis B virus-related cirrhosis. Indian J Gastroenterol. 2015; 34:286–91. https://doi.org/10.1007/s12664-015-0576-1 PMID:26243587

19. Hsu YC, Wu CY, Lane HY, Chang CY, Tai CM, Tseng CH, Lo GH, Perng DS, Lin JT, Mo LR. Determinants of hepatocellular carcinoma in cirrhotic patients treated with nucleos(t)ide analogues for chronic hepatitis B. J Antimicrob Chemother. 2014; 69:1920–27. https://doi.org/10.1093/jac/dku041 PMID:24576950

20. Hanumanthappa DK, Khan R, Wilkinson M, Wong T, Tritto G. Tenofovir and entecavir in the treatment of chronic hepatitis B: Effectiveness and safety in a single centre experience. Gut. 2014;63:A244. https://doi.org/10.1136/gutjnl-2014-307263.525

21. Coffin CS, Rezaeeaval M, Pang JX, Alcantara L, Klein P, Burak KW, Myers RP. The incidence of hepatocellular carcinoma is reduced in patients with chronic hepatitis B on long-term nucleos(t)ide analogue therapy. Aliment Pharmacol Ther. 2014; 40:1262–69. https://doi.org/10.1111/apt.12990 PMID:25312649

22. Köklü S, Tuna Y, Gülşen MT, Demir M, Köksal AŞ, Koçkar MC, Aygün C, Coban S, Ozdil K, Ataseven H, Akin E, Pürnak T, Yüksel I, et al. Long-term efficacy and safety of lamivudine, entecavir, and tenofovir for treatment of hepatitis B virus-related cirrhosis. Clin Gastroenterol Hepatol. 2013; 11:88–94. https://doi.org/10.1016/j.cgh.2012.10.003 PMID:23063679
