# Supplementary material for: No difference in hepatocellular carcinoma risk in chronic hepatitis B patients treated with tenofovir vs entecavir: evidence from an updated meta-analysis
Source: Aging (Albany NY). 2021 Feb 26;13(5):7147–65. doi: 10.18632/aging.202573 (PMC7993671; doi:10.18632/aging.202573)
Supplement: Supplementary Table 2 [file aging-13-202573-s003.docx]

**Supplementary Table 2. Main characteristics of included cohort studies.**

| Study; location | Sample size | Follow-up^*^, year | Age^*^, year | | Male, n (%) | | Cirrhosis, n (%) | | Positive HBeAg, n (%) | | HBV DNA^*^, log_10_ IU/ml | | Adjustment factors |
| --- | --- | --- | --- | --- | --- | --- | --- | --- | --- | --- | --- | --- | --- |
|  |  |  | TDF | ETV | TDF | ETV | TDF | ETV | TDF | ETV | TDF | ETV |  |
| Ha et al. (2020) [33]; Korea | 404 | 4.1^†^ in TDF group and 5.3^†^ in ETV group | 44.5 | 45.4 | 120 (53.6) | 106 (58.9) | 78 (34.8) | 67 (37.2) | 128 (57.1) | 118 (67.4) | 7.4^†^ | 7.7^†^ | None. |
| Hu et al. (2020) [32]; China | 894 | 5.0 | 56.1 | 59.4 | 162 (75.0) | 491 (72.4) | 216 (100.0) | 678 (100.0) | 41 (19.0) | 125 (18.4) | 7.4 | 7.1 | Platelet count, serum α-fetoprotein, diabetes, hepatocellular carcinoma family history, upper gastrointestinal varices, and propensity score ^¶^ |
| Su et al. (2020) [19]; USA | 3287 | 5.4 | 55.4 | 56.5 | 1039 (95.0) | 2116 (96.5) | 228 (20.8) | 454 (20.7) | 251 (22.9) | 664 (30.3) | 4.4^†^ | 4.9^†^ | Age, race, sex, body mass index, diabetes, Charlson comorbidity index, alcohol use disorder, HIV coinfection, HCV coinfection, cirrhosis, decompensated cirrhosis, receipt of prior antiviral therapy, HBV DNA, HBeAg status, platelet count, AST/ALT, albumin, bilirubin, international normalized ratio, creatinine |
| Shin et al. (2020) [31]; Korea | 1794 | 3.8^†^ in TDF group and 6.9^†^ in ETV group | 51.0 | 52.0 | 571 (63.4) | 597 (66.8) | 375 (41.7) | 440 (49.2) | 565 (62.8) | 537 (60.1) | 5.2^†^ | 6.5^†^ | Age, sex, treatment group, HBV DNA, HBeAg positivity, ALT, serum albumin, international normalized ratio, platelet count, diabetes mellitus, cirrhosis, virologic response at 12 months, and maintained virologic response |
| Papatheodoridis et al. (2020) [18]; Europe | 1935 | 7.1 | 53.0 | 52.0 | 827 (71.0) | 538 (70.0) | 358 (30.8) | 166 (21.5) | 233 (20.0) | 110 (14.2) | NA | NA | Age, sex, platelet count, nucleos(t)ide analogue treatment before TDF/ETV, and cirrhosis |
| Oh et al. (2020) [17]; Korea | 1560 | 4.7 | 46.3 | 48.7 | 503 (62.3) | 480 (63.7) | 310 (38.4) | 315 (41.8) | 484 (60.0) | 451 (61.4) | 6.6^†^ | 6.5^†^ | Age, sex, chronic kidney disease, diabetes, hypertension, cirrhosis, HBeAg, platelet count, albumin, total bilirubin, prothrombin time, serum α-fetoprotein, serum HBV DNA, Child-Turcotte-Pugh, and model for end-stage liver disease |
| Ha et al. (2020) [16]; Korea | 1340 | 4.5 | 45.0^†^ | 48.0^†^ | 266 (63.0) | 558 (61.0) | 39 (9.3) | 259 (28.0) | 261 (62.0) | 488 (53.0) | 6.7^†^ | 6.4^†^ | Age, sex, diabetes, cirrhosis, α-fetoprotein, albumin, and platelet count, |
| Lee et al. (2019) [10]; Korea | 3022 | 3.7 | 47.3 | 46.7 | 841 (58.4) | 926 (58.5) | 483 (33.6) | 567 (35.8) | 823 (57.2) | 974 (61.5) | 6.4^†^ | 6.5^†^ | Age, sex, severity of underlying liver disease, AST to platelet ratio index, fibrosis 4 index, diabetes, hypertension, body mass index, alcohol drinking, esophageal varix, AST, ALT, γ-GTT, total bilirubin, albumin, creatinine, prothrombin time, platelet counts, Child-Pugh score, HBeAg status, HBV DNA, and alpha-fetoprotein |
| Kim et al. (2019) [11]; Korea | 2897 | 4.9^†^ | 48.8 | 48.2 | 913 (64.6) | 889 (59.9) | 411 (29.1) | 499 (33.6) | 694 (49.1) | 758 (51.1) | 5.4 | 5.7 | Age, sex, diabetes, hypertension, compensated cirrhosis, HBeAg status, total bilirubin, albumin, and platelet counts |
| Hsu et al. (2019) [12]; USA, China, Japan, Korea | 5537 | 3.2^†^ in TDF group and 5.0^†^ in ETV group | 45.7 | 50.8 | 456 (65.1) | 3328 (68.8) | 131 (18.7) | 1475 (26.6) | 208 (29.7) | 1537 (31.8) | 5.0 | 5.5 | Age, sex, Asian countries, serum albumin, platelet count, alpha-fetoprotein, cirrhosis, and diabetes |
| Choi et al. (2019) [15]; Korea (nationwide cohort) | 24156 | 3.9 | 48.6 | 49.3 | 7949 (62.6) | 7171 (62.6) | 3488 (27.5) | 2991 (26.1) | NA | NA | NA | NA | Age, sex, socioeconomic status, level of health-care, cirrhosis, ascites, varices, diabetes, hypertension, smoking, drinking, body mass index, and ALT |
| Choi et al. (2019) [15]; Korea (validation hospital cohort) | 2701 | 3.0 | 48.1 | 49.2 | 692 (60.6) | 965 (61.9) | 653 (57.2) | 935 (59.9) | 641 (56.2) | 853 (54.7) | 6.4^†^ | 6.7^†^ | Age, sex, HBeAg status, HBV DNA, ALT, albumin, prothrombin time, platelet counts, diabetes, cirrhosis, ascites, varices, and virologic response |
| Yip et al. (2019) [14]; China | 29350 | 3.6^†^ | 43.2 | 53.4 | 591 (45.1) | 13263 (47.3) | 38 (2.9) | 3822 (13.6) | 721 (55.1) | 8317 (29.7) | 4.9 | 5.3 | Age, sex, cirrhosis, HBeAg positivity, ALT, albumin, platelet counts, and the calendar year of treatment initiation |
| Kim et al. (2018) [13]; Korea | 1325 | 3.4 | 50.0 | 52.0 | 363 (60.1) | 471 (65.3) | 267 (44.2) | 346 (48.0) | 376 (62.3) | 430 (59.7) | 6.0 | 6.4 | Age, sex, liver cirrhosis, platelet counts, and diabetes |

Abbreviations: TDF, tenofovir; ETV, entecavir; NA, not available; ALT, alanine aminotransferase; AST, aspartate aminotransferase.

^*^ Mean value unless otherwise specified.

^†^ Median value.

^¶^ Propensity score was calculated from logistic regression model including the following variables: age, serum α-fetoprotein, albumin, HBV DNA, estimated Glomerular filtration rate, hepatocellular carcinoma family history, and upper gastrointestinal varices.
