# Supplementary material for: No difference in hepatocellular carcinoma risk in chronic hepatitis B patients treated with tenofovir vs entecavir: evidence from an updated meta-analysis
Source: Aging (Albany NY). 2021 Feb 26;13(5):7147–65. doi: 10.18632/aging.202573 (PMC7993671; doi:10.18632/aging.202573)
Supplement: Supplementary Table 3 [file aging-13-202573-s004.pdf]

SUPPLEMENTARY TABLE

Supplementary Table 3. Results of quality assessment of included studies.

	Selection				Comparability	Outcome			
**Study**	**Representativeness of exposed cohort☆**	**Selection of non-exposed cohort☆**	**Exposure ascertainment ☆**	**No history of disease☆**	**Comparable on confounders☆☆**	**Outcome assessment☆**	**Adequate follow-up (≥10y)☆**	**Loss to follow-up rate (≤20%) ☆**	**Total score**
Ha et al. (2020) [33]		☆	☆	☆	☆	☆		☆	6
Hu et al. (2020) [32]		☆	☆	☆	☆	☆		☆	6
Su et al. (2020) [19]	☆	☆	☆	☆	☆ ☆	☆		☆	8
Shin et al. (2020) [31]	☆	☆	☆	☆	☆ ☆	☆		☆	8
Papatheodoridis et al. (2020) [18]	☆	☆	☆	☆	☆ ☆			☆	7
Oh et al. (2020) [17]		☆	☆	☆	☆ ☆	☆		☆	7
Ha et al. (2002) [16]		☆	☆	☆	☆	☆		☆	6
Lee et al. (2019) [10]		☆	☆	☆	☆ ☆	☆		☆	7
Kim et al. (2019) [11]		☆	☆	☆	☆	☆		☆	6
Hsu et al. (2019) [12]	☆	☆	☆	☆	☆	☆		☆	7
Choi et al. (nationwide cohort) (2019) [15]	☆	☆	☆	☆	☆ ☆	☆		☆	8
Choi et al. (validation hospital cohort) (2019) [15]		☆	☆	☆	☆ ☆	☆		☆	7
Yip et al. (2019) [14]	☆	☆	☆	☆	☆ ☆	☆		☆	8
Kim et al. (2018) [13]		☆	☆	☆	☆	☆		☆	6

Note that the reference numbers refer to the reference list in the main article.
