# Supplementary material for: No difference in hepatocellular carcinoma risk in chronic hepatitis B patients treated with tenofovir vs entecavir: evidence from an updated meta-analysis
Source: Aging (Albany NY). 2021 Feb 26;13(5):7147–65. doi: 10.18632/aging.202573 (PMC7993671; doi:10.18632/aging.202573)
Supplement: Supplementary Table 4 [file aging-13-202573-s005.docx]

**Supplementary Table 4. Main characteristics of included studies after propensity score matching.**

| Study | Number of matched pairs | Age^*^, year | | Male, n (%) | | Cirrhosis, n (%) | | Positive HBeAg, n (%) | | HBV-DNA^*^, log_10_ IU/ml | | Variables used for propensity score matching |
| --- | --- | --- | --- | --- | --- | --- | --- | --- | --- | --- | --- | --- |
|  |  | TDF | ETV | TDF | ETV | TDF | ETV | TDF | ETV | TDF | ETV |  |
| Ha et al. (2020) [33] | 168 | 45.0 | 45.4 | 94 (56.0) | 100 (59.5) | 56 (33.3) | 58 (34.5) | 109 (64.9) | 111 (66.1) | 7.7^†^ | 7.8^†^ | Age, sex, cirrhosis, diabetes, HBeAg, HBV DNA, platelet count, albumin, total bilirubin, prothrombin time, ALT, creatinine, Child–Pugh score, GAG-hepatocellular carcinoma score, Chinese University-hepatocellular carcinoma score, PAGE-B score, and sustained virological suppression |
| Shin et al. (2020) [31] | 589 | 50.0 | 50.0 | 358 (60.8) | 365 (62.0) | 282 (47.9) | 276 (46.9) | 354 (60.1) | 365 (62.0) | 6.2^†^ | 6.1^†^ | Age, HBV DNA, ALT, albumin, total bilirubin, creatinine, platelet count, diabetes, hypertension, and liver cirrhosis |
| Oh et al. (2020) [17] | 516 | 49.0 | 49.2 | 325 (63.0) | 319 (61.8) | 224 (43.4) | 238 (46.1) | 311 (60.3) | 314 (60.9) | 6.4^†^ | 6.4^†^ | Age, sex, chronic kidney disease, diabetes, hypertension, cirrhosis, decompensated status, HBeAg, HBV DNA, Child-Turcotte-Pugh, model for end-stage liver disease, FIB-4 index, serum α-fetoprotein, platelet count, albumin, total bilirubin, estimated glomerular filtration ratio, and prothrombin time |
| Ha et al. (2002) [16] | 298 | 48.0^†^ | 48.0^†^ | 179 (60.0) | 181 (61.0) | 39 (9.0) | 39 (9.0) | 174 (58.0) | 161 (54.0) | 6.3^†^ | 6.4^†^ | Age, sex, drinking history, diabetes, cirrhosis, HBeAg positivity, HBV DNA, HBsAg titer, AST, ALT, α-fetoprotein, albumin, bilirubin, prothrombin time, platelet count, and calendar year of treatment initiation |
| Lee et al. (2019) [10] | 1370 | 46.9 | 47.0 | 798 (58.3) | 806 (58.8) | 464 (33.9) | 465 (33.9) | 807 (58.9) | 814 (59.4) | 6.4^†^ | 6.5^†^ | Age, sex, severity of underlying liver disease, APRI, FIB-4 index, diabetes, hypertension, body mass index, alcohol drinking, esophageal varix, AST, ALT, total bilirubin, albumin, creatinine, γ-GTT, prothrombin time, platelet count, Child-Pugh score, HBeAg status, HBV DNA, and AFP |
| Kim et al. (2019) [11] | 1278 | 48.2 | 48.6 | 794 (62.1) | 793 (62.1) | 411 (29.1) | 499 (33.6) | 694 (49.1) | 758 (51.1) | 5.4 | 5.7 | Age, sex, diabetes, hypertension, compensated cirrhosis, HBeAg status, total bilirubin, albumin, and platelet counts |
| Hsu et al. (2019) [12] | 520 | 44.9 | 44.1 | 338 (65.0) | 354 (68.1) | 105 (20.2) | 107 (20.6) | 177 (34.0) | 187 (36.0) | 5.1 | 5.0 | Age, sex, country of study centers, cirrhosis, diabetes, HBeAg status, HBV DNA, ALT, platelet counts, and hepatic decompensation |
| Choi et al. (2019) [15] (nationwide cohort) | 10923 | 49.0 | 49.1 | 6834 (62.6) | 6802 (62.3) | 2919 (26.7) | 2891 (26.5) | NA | NA | NA | NA | Age, sex, socioeconomic status, level of health care, smoking, cirrhosis, diabetes, and hypertension |
| Choi et al. (2019) [15] (validation hospital cohort) | 869 | 48.8 | 48.8 | 540 (62.1) | 519 (59.7) | 505 (58.1) | 511 (58.8) | 481 (55.4) | 479 (55.1) | 6.5^†^ | 6.5^†^ | Age, sex, HBeAg positivity, HBV DNA levels, ALT, albumin, total bilirubin, international normalized ratio, platelet count, creatinine concentration, diabetes, hypertension, cirrhosis, ascites, Child-Pugh score, Chinese University HCC score, *Guide With Age, Gender, HBV DNA, Core Promoter Mutations, and Cirrhosis–HCC* score, Platelet Age Gender B score, and Risk Estimation for Hepatocellular Carcinoma in Chronic Hepatitis B score |
| Yip et al. (2019) [14] | 1200 | 44.4 | 42.9 | 587 (48.9) | 587 (48.9) | 37 (3.1) | 43 (3.6) | 625 (52.1) | 642 (53.5) | 4.8 | 4.8 | Age, sex, HBeAg positivity, HBV DNA levels, ALT, albumin, total bilirubin, international normalized ratio, platelet count, creatinine, renal replacement therapy, cirrhosis, ascites, hepatic encephalopathy, diabetes, hypertension, calendar year of treatment initiation |
| Kim et al. (2018) [13] | 354 | 51.0 | 51.0 | 222 (62.7) | 220 (62.1) | 156 (44.1) | 169 (47.7) | 223 (63.0) | 232 (65.5) | 6.2 | 6.2 | Age, sex, HBeAg positivity, cirrhosis, HBV DNA levels, ALT, AST, albumin, total bilirubin, creatinine, alpha-fetoprotein, international normalized ratio, platelet count, diabetes, and hypertension |

Abbreviations: TDF, tenofovir; ETV, entecavir; NA, not available; ALT, alanine aminotransferase; AST, aspartate aminotransferase.

^*^ Mean value unless otherwise specified.

^†^ Median value.
