# Supplementary material for: No difference in hepatocellular carcinoma risk in chronic hepatitis B patients treated with tenofovir vs entecavir: evidence from an updated meta-analysis
Source: Aging (Albany NY). 2021 Feb 26;13(5):7147–65. doi: 10.18632/aging.202573 (PMC7993671; doi:10.18632/aging.202573)
Supplement: Supplementary Tables 5 and 6 [file aging-13-202573-s006.pdf]

SUPPLEMENTARY TABLES

Supplementary Table 5. Sensitivity analyses on the comparative effectiveness of tenofovir versus entecavir for hepatocellular carcinoma risk¶.

Eligibility criteria	N	HR (95% CI)	I^2 (%)
Excluding studies with sample size of >10000	9	0.95 (0.83–1.08)	5.4
Excluding studies with sample size of <2000	7	0.88 (0.72–1.07)	66.1
Restricting analysis to studies with the proportion of males between 35% to 65%	9	0.83 (0.67–1.03)	68.3
Excluding studies with quality score of <7 points	9	0.84 (0.63–1.05)	61.8
Excluding studies with follow-up length difference between two groups of >1 year	8	0.86 (0.68–1.08)	73.8
Excluding studies with mean or median age of patients of >50 years	6	0.79 (0.60–1.04)	73.2

Abbreviations: HR, hazard ratio; CI, confidence interval.
¶ All sensitivity analyses were based on multivariable-adjusted risk estimates.

Supplementary Table 6. Search Strategies for PubMed and EMBASE databases.

6.1 Search strategies for PubMed database (from its inception to November 24, 2020)

No.	Search strategy	Items found
#1	(((((cancer*[Title/Abstract]) OR neoplasm*[Title/Abstract]) OR carcinoma*[Title/Abstract]) OR malignanc*[Title/Abstract])) AND (((liver[Title/Abstract]) OR hepatic[Title/Abstract]) OR hepatocellular[Title/Abstract])	202752
#2	((((carcinoma, hepatocellular[MeSH Terms]) OR liver neoplasms[MeSH Terms]) OR hepatocarcinoma[Title/Abstract]) OR "liver cell carcinoma*"[Title/Abstract]) OR hepatoma[Title/Abstract]	179081
#3	#1 OR #2	281483
#4	((entecavir[Supplementary Concept]) OR Baraclude[Title/Abstract]) OR entecavir[Title/Abstract]	2762
#5	((((tenofovir[MeSH Terms]) OR tenofovir[Title/Abstract]) OR Viread[Title/Abstract]) OR Vemlidy[Supplementary Concept]) OR Vemlidy[Title/Abstract]	7725
#6	#3 AND #4 AND #5	323

6.2 Search strategies for EMBASE database (from its inception to November 24, 2020)

No.	Search strategy	Items found
#1	cancer*:ab,ti OR neoplasm*:ab,ti OR carcinoma*:ab,ti OR malignanc*:ab,ti	3390750
#2	liver:ab,ti OR hepatic:ab,ti OR hepatocellular:ab,ti	1333482
#3	#1 AND #2	297271
#4	'liver cell carcinoma'/exp OR 'liver cancer'/exp OR hepatocarcinoma:ab,ti OR 'liver cell carcinoma*':ab,ti OR hepatoma:ab,ti	280560
#5	#3 OR #4	400476
#6	'entecavir'/exp OR baraclude:ab,ti OR entecavir:ab,ti	9149
#7	'tenofovir'/exp OR tenofovir:ab,ti OR viread:ab,ti OR 'tenofovir alafenamide'/exp OR vemlidy:ab,ti	23334
#8	#5 AND #6 AND #7	1285
